# Interactive molecular dynamics in virtual reality for modelling materials and catalysts

**DOI:** 10.1016/j.jmgm.2023.108606

**Published:** 2023-08-24

**Authors:** Joe Crossley-Lewis, Josh Dunn, Corneliu Buda, Glenn J. Sunley, Alin M. Elena, Ilian T. Todorov, Chin W. Yong, David R. Glowacki, Adrian J. Mulholland, Neil L. Allan

**Affiliations:** a Centre for Computational Chemistry, School of Chemistry, University of Bristol, Cantock’s Close, Bristol, BS8 1TS, UK; b Applied Sciences, bp Innovation and Engineering, BP plc, 150 West Warrenville Road, Naperville, IL, 60563, USA; c Applied Sciences, bp Innovation and Engineering, BP plc, Saltend, Hull, HU12 8DS, UK; d Scientific Computing Department, STFC Daresbury Laboratory, Daresbury, UK

**Keywords:** Virtual reality, Materials, Molecular dynamics, Interactive molecular dynamics in virtual, reality zeolite catalysis, Defects, Fast-ion conduction

## Abstract

Interactive molecular dynamics simulation in virtual reality (iMD-VR) is emerging as a promising technique in molecular science. Here, we demonstrate its use in a range of fifteen applications in materials science and heterogeneous catalysis. In this work, the iMD-VR package Narupa is used with the MD package, DL_POLY [1]. We show how iMD-VR can be used to: (i) investigate the mechanism of lithium fast ion conduction by directing the formation of defects showing that vacancy transport is favoured over interstitialcy mechanisms, and (ii) guide a molecule through a zeolite pore to explore diffusion within zeolites, examining in detail the motion of methyl *n*-hexanoate in H-ZSM-5 zeolite and identifying bottlenecks restricting diffusion. iMD-VR allows users to manipulate these systems intuitively, to drive changes in them and observe the resulting changes in structure and dynamics. We make these simulations available, as a resource for both teaching and research. All simulation files, with videos, can be found online (https://doi.org/10.5281/zenodo.8252314) and are provided as open-source material.

## 1 Introduction

Atomic-level simulations are playing an ever-increasing role in materials chemistry and condensed matter physics, both in research and in education [1–6]. They can identify structural features relating to activity, and it is possible to control and analyse simulations in ways and at a level of detail no experiment can reach so far. Such simulations can present a testbed of possibilities and act as a source of ideas and means of testing hypotheses. They can help experimentalists and technologists to identify promising systems and probe their properties. Such simulations are now an integral part of materials design, with applications in important areas such as catalysis for sustainability, pollutant removal, battery development, and renewable energy [7–9]. Simulations are still, typically the domain of the expert computational scientist. There is a need to make them more accessible to non-specialists, and to integrate them with experimental studies.

**Fig. 1:**
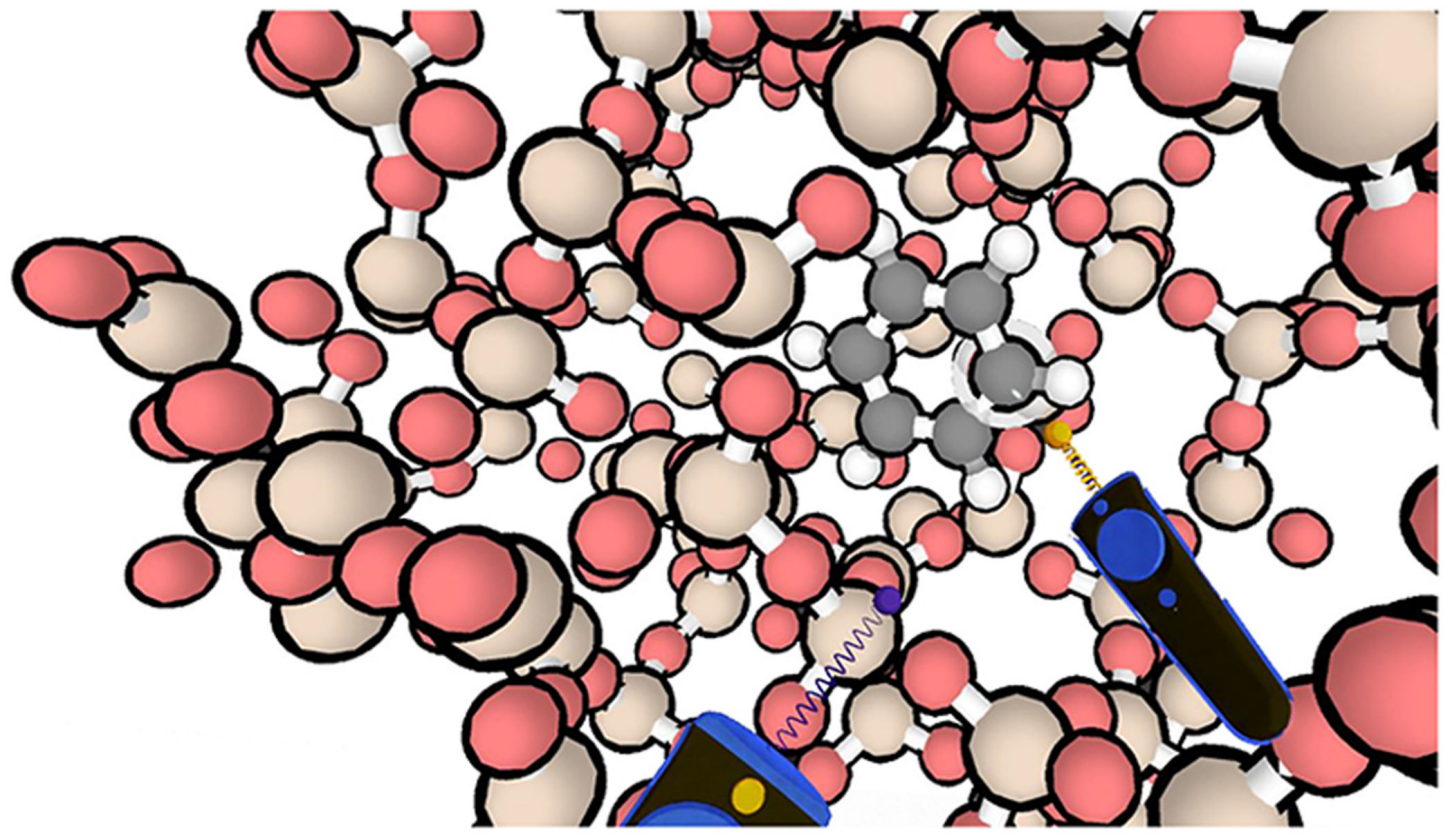
H-ZSM-5 zeolite with a benzene molecule, showing the user view in iMD-VR. The Narupa controllers can be seen interacting with the benzene, manipulating the position of the molecule within the zeolite framework.

In computational *molecular* science, interactive simulation methods in virtual reality (VR) are emerging as transformative technology [10–18]. The potential of VR to provide insight into three-dimensional structures has long been recognised [19–23], but *interactive* simulations in VR go further in allowing not only visualisation of structures, but also the manipulation of their dynamics (Fig. 1). Interactive molecular dynamics in VR (iMD-VR) offers an accessible way for users to interact with the atomic-world and accelerate molecular simulations ‘on the fly’ using human intuition.

A challenge in standard MD simulations is that processes involving significant energy barriers may occur rarely or not at all, even with simulation times of micro/milliseconds. Rare events can be simulated by enhanced sampling techniques e.g. involving the addition of a biasing potential [24]. Such enhanced sampling MD methods include umbrella sampling, conformational flooding, steered MD, metadynamics, and variational enhanced sampling [25–31]. Interactive molecular dynamics (iMD) also allows enhanced sampling, by allowing users to manipulate the system directly and traverse barriers by applying force to it. iMD consists of a simulation run by an MD package that is linked to a 3D visualisation engine; the user(s) interacts with the simulation in real time using input devices. In iMD-VR, virtual reality is used to visualise the 3D structures and the user interacts with the simulation using handheld controllers as shown in Fig. 1 [32–41]. Perhaps most importantly, iMD-VR allows human intuition and knowledge to be brought to bear on molecular problems in an accessible way, while also being based on fundamental physics of Newtonian dynamics.

iMD-VR offers new approaches for collaboration: many users can participate in the same virtual space and interact with the same system. With appropriate high-speed networking, users can work together in the same virtual environment while in different physical locations. The need for tools to facilitate virtual collaboration has been highlighted by the COVID-19 pandemic. iMD-VR has been applied in areas ranging from modelling molecular transport in zeolites to protein-ligand docking [10, 42], including a study of the SARS-Cov-2 main protease [43], to teaching undergraduate students about enzyme catalysis [1]. These include applications in which several users participate in the same virtual space.

In chemistry and materials science, 3D spatial reasoning is key to concepts such as symmetry, crystallography, and stereochemistry [44, 45]. Contemporary GUIs such as PyMol, visual molecular dynamics (VMD), GaussView, Samson and VESTA allow visualisation, animation, and interpretation of structures of up to hundreds of thousands of atoms [46–50]. These programs generally require high-level technical proficiency and can require knowledge of domain-specific input languages or syntaxes, resulting in learning and usage barriers. Limitations also arise due to visual perception of 3D molecular structures using 2D tools [12]. Demand for immersive 3D visualisation and intuitive interaction for chemical applications has resulted in the development of tools such as the CAVE automatic virtual environment [51], force haptic probes [52], augmented reality apps [13], and Haptic Quantum Chemistry [53]. High quality, increasingly affordable VR equipment has allowed 3D visualisation to become more widely available [54]. This work uses a typical VR set-up consisting of a head-mounted display, infrared active base station trackers and controllers. Modern VR hardware supports six degrees of freedom – three corresponding to rotational axis movement and three corresponding to translational axis movement. Rapid refresh rates, and other factors such as improvements to software lag and focal rendering techniques, have reduced considerably the historical problem of motion sickness experienced in VR [54].

The benefits of using iMD-VR have been quantified by O’Connor et al. [10] through a series of human computer interaction studies. iMD-VR provided a clear advantage in complex 3D tasks. Statistical analysis of controlled human-computer interaction studies showed that participants complete molecular modelling tasks more quickly and more efficiently when using an iMD-VR framework, compared to using a traditional mouse/screen interface [1]. The user can drive the simulation, helped by their chemical intuition and understand directly how the system evolves dynamically [55].

In this work, we show how iMD-VR can also be used effectively to investigate and manipulate condensed phases in materials science and solid-state chemistry. Examples include the use of iMD-VR to drive atomic migration and ionic conduction, and to probe molecular transport in microporous solids. All of the examples here use molecular mechanics forcefields via the DL-POLY code [56].

## 2 Methods

### 2.1 Software

**Scheme 1:**
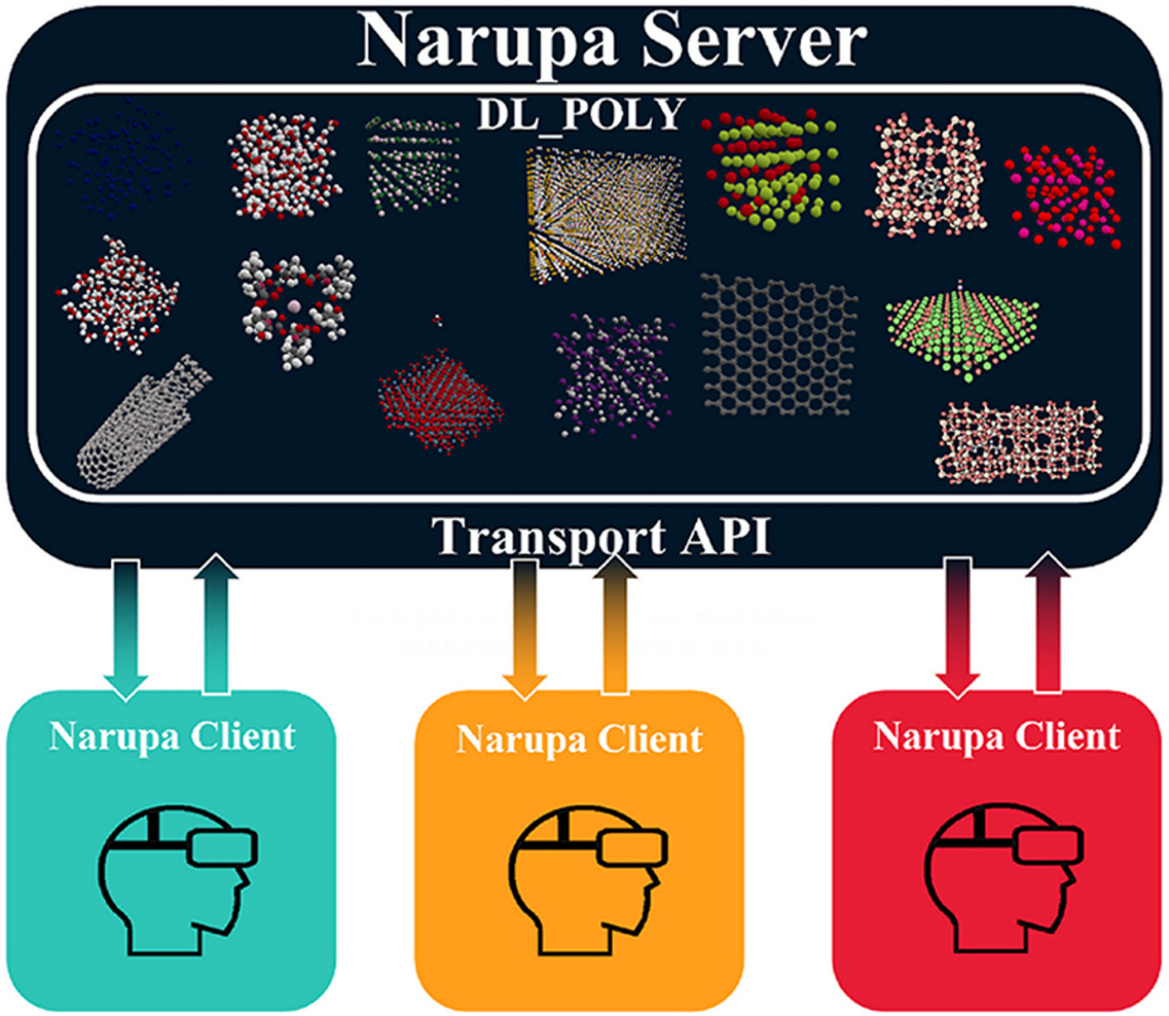
Outline of the client/server design of Narupa with the API that enables the communication between the VR client and the MD server. The different coloured Narupa clients indicate different distant VR users.

The iMD-VR package Narupa [10,57] was linked to the DL_POLY MD package (version 4.09.4) with the open-source PLUMED library (version 2.5.0) [56,58]. An application programming interface (API) communicates between the VR client and the simulation server; multiple clients may be set up simultaneously as illustrated in Scheme 1 to allow for multi-user iMD-VR. In the Narupa iMD-VR package [10,57], the choice of simulation server is not limited to those described in this work: other force engines have been implemented, including OpenMM [59], LAMMPS [60], the tight binding density functional theory package DFTB+ [61,62], and the semiempirical quantum chemistry package SCINE [63]. The API sends the atomic positions to a force engine and receives forces in return [10,57]. The Narupa client converts these data into a visualisation of the simulation that also allows user interaction. Forces are sent back to the simulation server, including user interaction forces, thus creating an iMD-VR framework.

### 2.2 Hardware

Narupa is compatible with the Oculus Rift, HTC VIVE, HTC VIVE Pro, and the Valve Index VR headsets [57]. The work presented here used the Valve Index on an Ubuntu 18.04 WSL1 Linux subsystem on a Microsoft Windows 10 operating system. The VR software runs natively on a Windows system and the simulation engine, DL_POLY runs on a Linux system. The VR equipment used here required a headset, left- and right-handed controllers, and two mountable IR transmitters that translate physical movement into the VR environment. A GPU is required to use the headset with the requirement of a minimum graphic equivalent to the Nvidia GeForce GTX 970/AMD RX480. The workstation used for all the simulations in this manuscript was a Dell Precision 3630 computer with an NVIDIA GeForce GTX 1080 GPU and 8 GB GDDR5X RAM. The computer was equipped with an Intel Core i7 CPU, 16 GB RAM, 512 GB SSD.

### 2.3 Systems studied

**Fig. 2:**
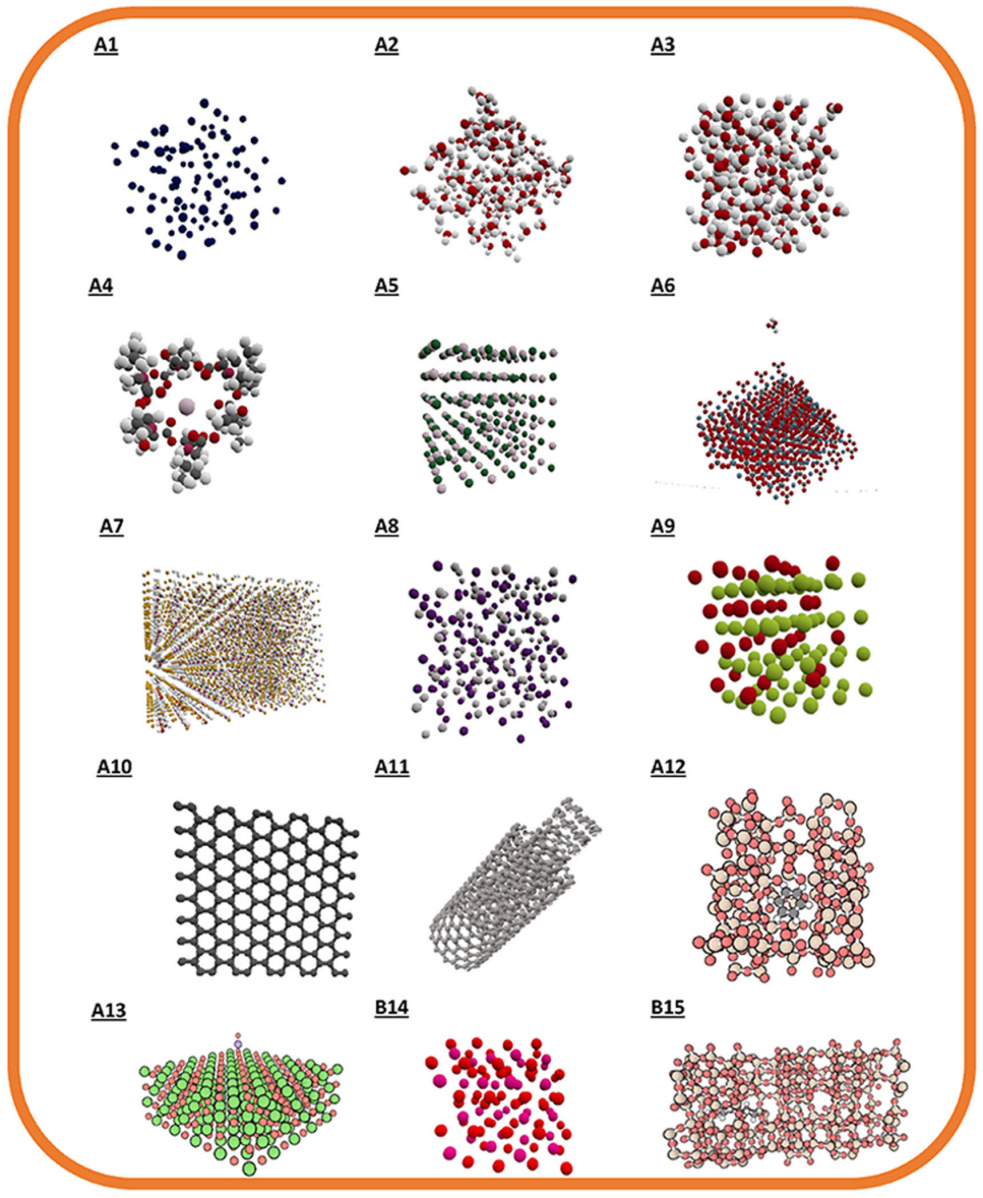
The materials systems studied here. Systems A1 to A13 focus on the qualitative benefits of using iMD-VR and the descriptions can be found in the SI. Systems B14 and B15 focus on more quantitative uses of iMD-VR and are explored in the results and discussion section. From A1 to B15 the systems are: liquid argon, single point charge (SPC) water, a potassium ion in SPC water, potassium and valinomycin, potassium chloride, formic acid on a calcite surface, silver iodide, zirconia, graphene, a carbon nanotube switch, benzene in ZSM-5 zeolite, a barium oxide pair on a barium oxide surface, lithium oxide, methyl *n*-hexanoate in HZSM-5 zeolite.

All fifteen systems investigated here are shown in Fig. 2. A short description of each system, including a link to a video of the system in iMD-VR, can be found in Table 1. The systems, selected both from the DL_POLY tutorials, and more widely from the literature, were chosen to represent a cross-section of structures and physical behaviour in materials chemistry relevant to industry and teaching, and to explore the uses of iMD-VR.

All simulation files, and videos, can be found online (https://doi.org/10.5281/zenodo.8252314) and are provided as open-source material. Trajectory files are also online for the Li_2_O and zeolite examples which are discussed in more depth. A short description of systems **A1** to **A13**, which concentrate on visualisation and the qualitative advantages of using iMD-VR compared to MD, can be found in the supplementary information; while examples **B14** to **B15** are explored in more detail in the results and discussion section below.

## 3 Results and discussion

### 3.1 Fast ion conduction in Li_2_O

The antifluorite crystal structure of lithium oxide makes for a good testbed for iMD-VR investigations of ion transport and fast-ion conduction because it undergoes a superionic phase transition (*T*_*C*_ » 1200–1350 K), above which Li^+^ ions are mobile and diffusion is rapid, before melting at 1700 K [64]. The oxide anions form a face-centred sublattice and the Li^+^ cations occupy all the tetrahedral holes forming a simple cubic lattice. The Li^+^ diffusion mechanisms have been the subject of debate [65–67]. Here, we use iMD-VR to compare Li^+^ migration via vacancies and interstitials.

A cubic simulation box (supercell) of 324 ions was used in the iMD-VR studies. This is small by modern simulation standards, but a larger cell is not feasible due to the current intensive computing requirements of simulation for real-time iMD-VR due to the MPI communication between the simulation server and VR client with DL_POLY. The simulations used two-body interionic Buckingham potentials and charges from work by Lavrentiev et al. [8] and Fracchia et al. [68] The simulations were run in the isothermal-isobaric (NPT) ensemble, using the Nosé–Hoover thermostat and barostat, with relaxation times of 0.05 and 0.25 ps, respectively [69]. Equilibration was carried out before production runs, this consisted of 10 ps of NVT simulation, as in Fracchia et al. [68] All production simulations were carried out at ambient pressure; run both with and without user interaction, at seven temperatures from 1000 to 1800 K, three runs each, with an integration timestep of 0.1 fs, for a total simulation time of 100 ps.

To examine the lithium transport mechanisms, a lithium Frenkel defect was first created in the Li_2_O simulation cell, as in Lavrentiev et al. [8], by removing a Li^+^ cation from the corner of the simulation cell and placing it into an interstitial position in an octahedral hole, *i.e*., the centre of a cube formed by six cations, close to the centre of the supercell [8]. This leaves a vacancy in the Li sublattice. Using iMD-VR, the nearest neighbour Li^+^ was readily moved into this vacancy. The simulations were then continued with no further interaction, until 100 ps of simulation time had elapsed (https://vimeo.com/611488899). If instead an interaction is applied to an interstitial Li, independent of direction of application, the interstitial ion jumps into the position of a neighbouring Li and the displaced cation then moves into another interstitial position. Direct interstitial migration through the crystal was not observed. This suggests the favoured mechanism for interstitial migration is an interstitialcy mechanism, in which one interstitial Li^+^ ion displaces an Li^+^ from a lattice site which in turn itself becomes an interstitial. It is also worth noting that the force required to initiate interstitial transport via this mechanism is larger than that to drive vacancy migration, consistent with the Monte Carlo simulations of Lavrentiev et al. [8] in which interstitialcy migration was only observed at approximately 900–1000 K, 300 K higher than the temperature at which the vacancy mechanism activates. Although applied forces were not measured directly here, they are apparent to the user due to increased interaction scaling factors and effort used in iMD-VR.

**Fig. 3:**
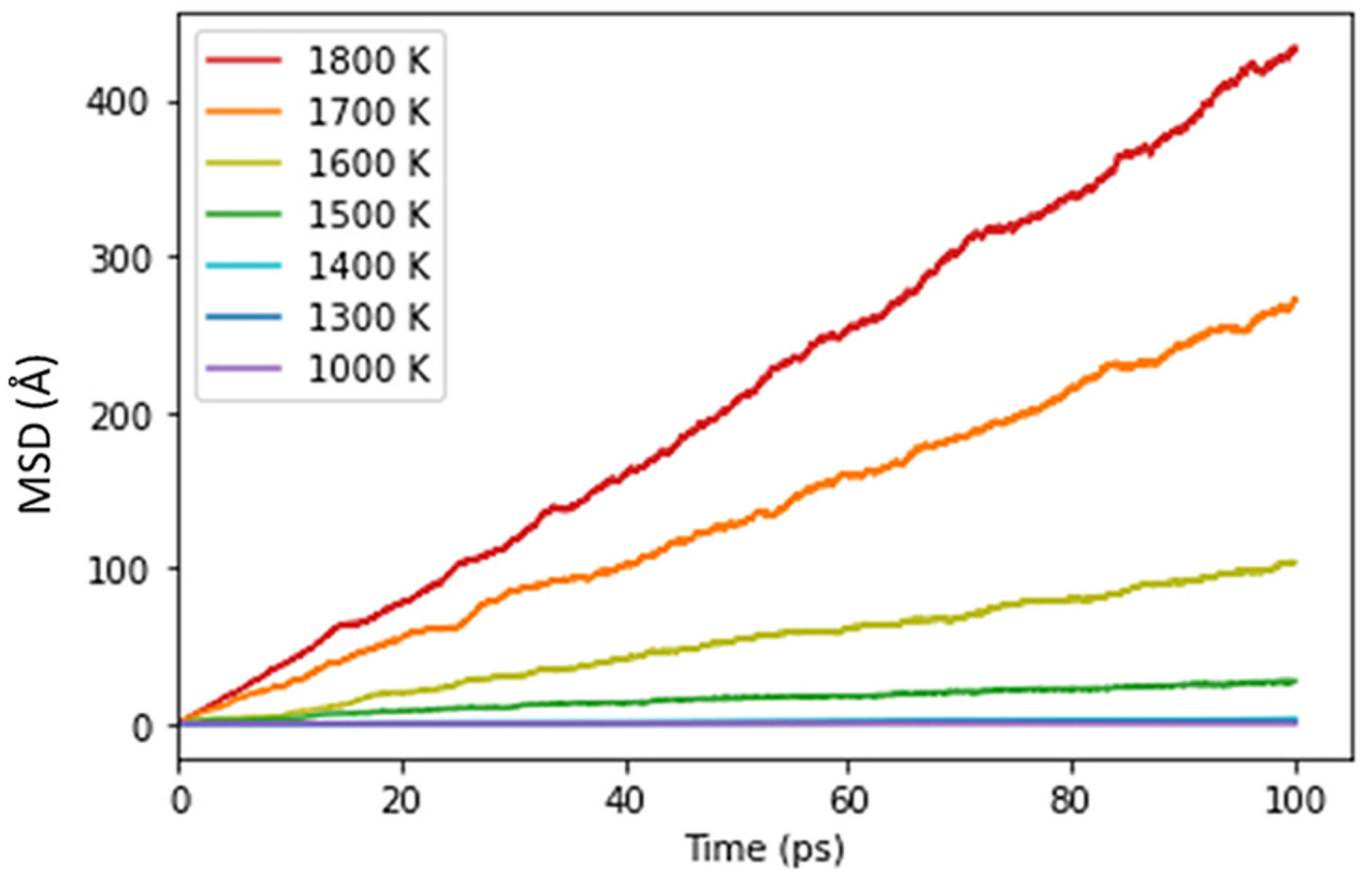
Mean-squared displacement over time of the Li ions in a 324 ion simulation cell of Li_2_O at different temperatures in the absence of any user interaction (i.e. during standard MD simulation). The increase in diffusion with temperature can be seen in the increased rate of change in MSD.

To check that the user interaction with the simulation did not move the system into an unphysical and experimentally inaccessible high-energy region of the energy landscape, the motion of the Li^+^ ions in the two types of simulation was compared. The mean square displacements (MSDs), <*r*^2^>, of the Li^+^ ions, were monitored during all simulations and the temperature-dependent self-diffusion coefficient, *D*_s_, determined from the Einstein relation (<*r*^2^> = 6*D*_s_*t*). For example, the MSD for simulations with no user interaction is plotted in Fig. 3. In passing, we note that in all the simulations, the oxide ions remained on their lattice sites: as expected in the superionic regime, transport is confined to the lithium ions.

**Fig. 4:**
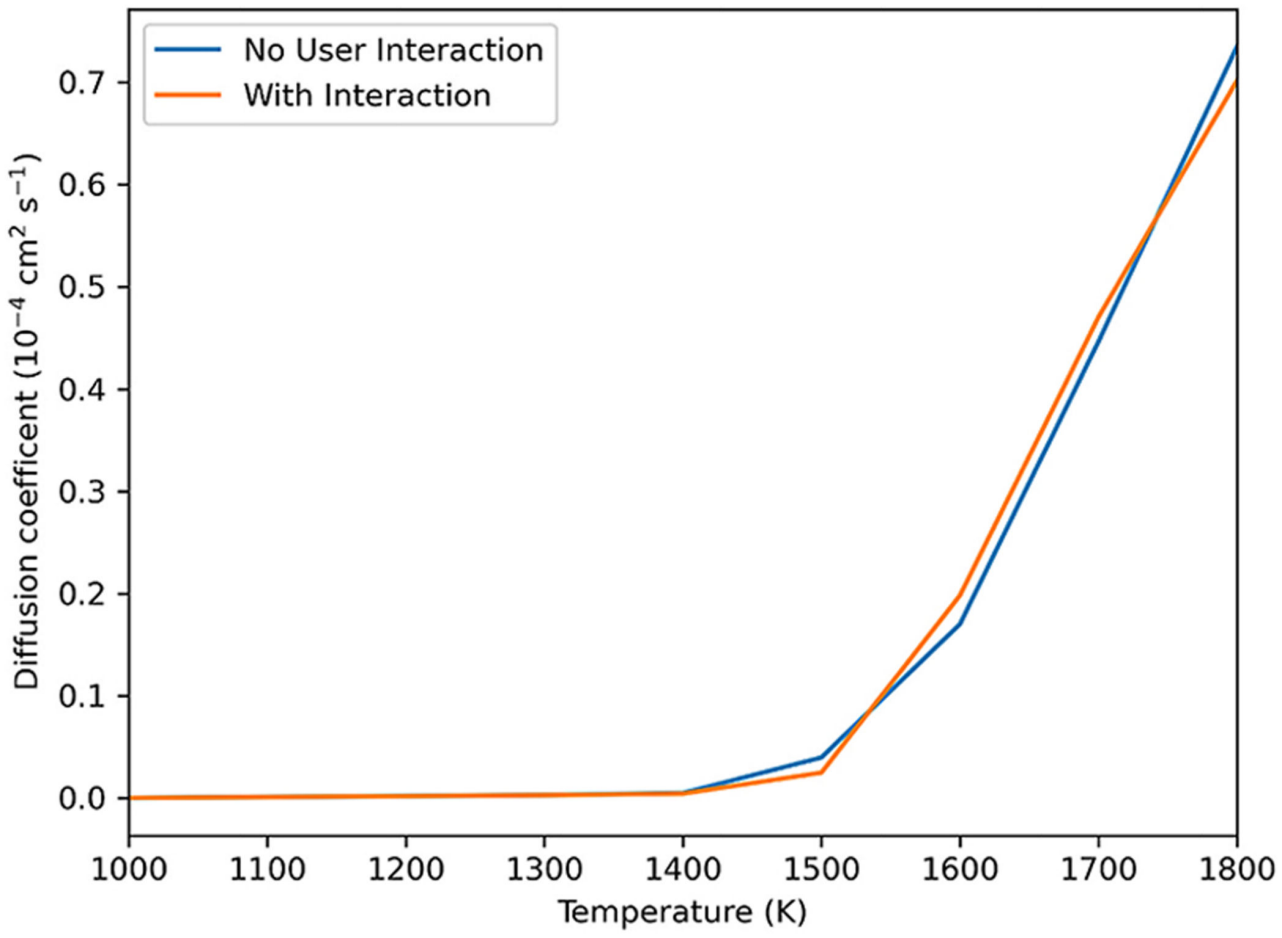
Diffusion coefficient at different temperatures for a 324 ion simulation cell of Li_2_O. There is no significant difference is seen between MD simulations with and without user interaction, showing that user interaction has not caused unphysical perturbation of the system.

**Fig. 5:**
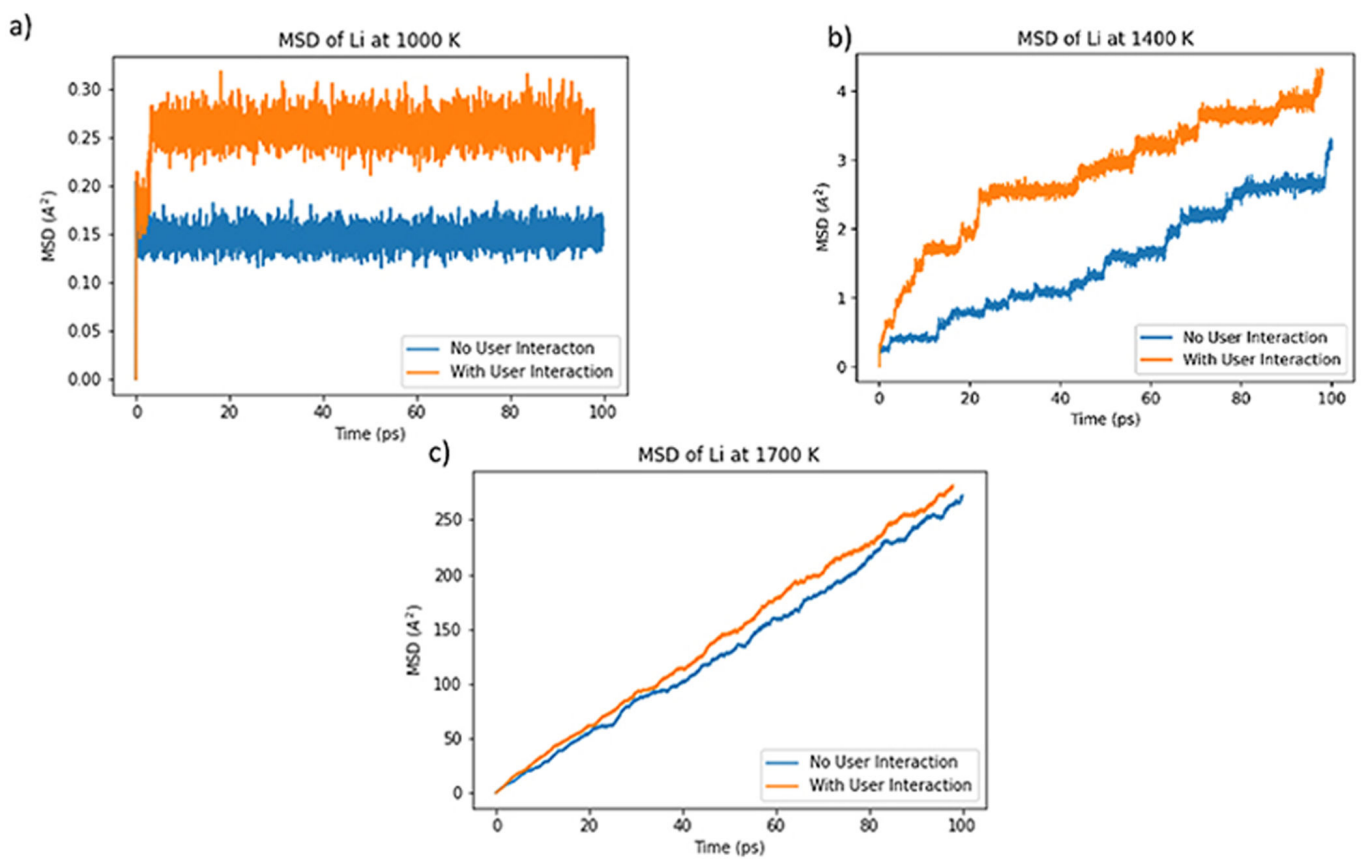
The mean-squared displacement of Li ions in a 324 ion Li_2_O simulation, with and without user interaction at a) 1000 K b) 1400 K and c) 1700 K. Although the values in these graphs differ, the gradients of the plots with and without user interaction are similar.

Fig. 4 shows that there is no significant difference between values of the self-diffusion coefficient *D*_s_ at all temperatures studied in simulations with and without user interaction. The vacancy follows a similar path through the lattice with or without user interaction but movement along such paths is accelerated by user interaction as seen in Fig. 5. While Fig. 5 demonstrates a difference between the MSDs with and without user interaction, the important feature is that the gradients of the plots in Fig. 5b and 5c are similar after the interaction period, and hence so are the diffusion coefficients. MSD plots for the oxide ions can be found in the Supplementary Information.

This shows that iMD-VR allows mechanisms of ion transport to be explored quickly, with immediate feedback. Here, we have shown that iMD-VR can probe the defect and transport properties of a fast-ion conductor such as Li_2_O, providing insights comparable to those from traditional extensive molecular simulations and subsequent analysis. We have also demonstrated explicitly that the user applied force is not large enough to bias the results significantly towards unphysical regions of the energy landscape.

### 3.2 Transport of methyl n-hexanoate through H-ZSM-5 zeolite

Organic carbonyl compounds have been found to significantly improve the catalytic efficiency of zeolites for the widely studied methanol to dimethyl ether (DME) dehydration reaction [70]. In particular the addition of methyl carboxylate esters was found to promote the formation of DME at low temperatures (110–150 °C) at concentrations as low as 0.001 mol % relative to methanol [70]. One such example of a promoter, methyl *n*-hexanoate, adsorbs strongly on Brønsted acid sites. Such sites arise when a proton is covalently bound to an oxygen atom adjacent to an aluminium atom, thus maintaining charge neutrality in the zeolite.

Diffusion is critical to catalysis within zeolites and it is often slow and rate-limiting [71–73]. Transport processes within zeolites often exist in single-file conditions due to the relative sizes of the pores and the molecules within them [74]. Under these conditions, promotion of preferential diffusion pathways may improve diffusion rates, and thus catalytic activity. This was first suggested by Derouane et al. [75] who coined the term ‘molecular traffic control’ [76,77]. Here, iMD-VR is used to explore the transport of the promoter methyl *n*-hexanoate in the H-ZSM-5 zeolite (https://vimeo.com/456311091). The promoter is unlikely to desorb over the typical timescales of a MD simulation and so iMD-VR is used to access the longer timescales needed to see the promoter diffusing.

**Fig. 6:**
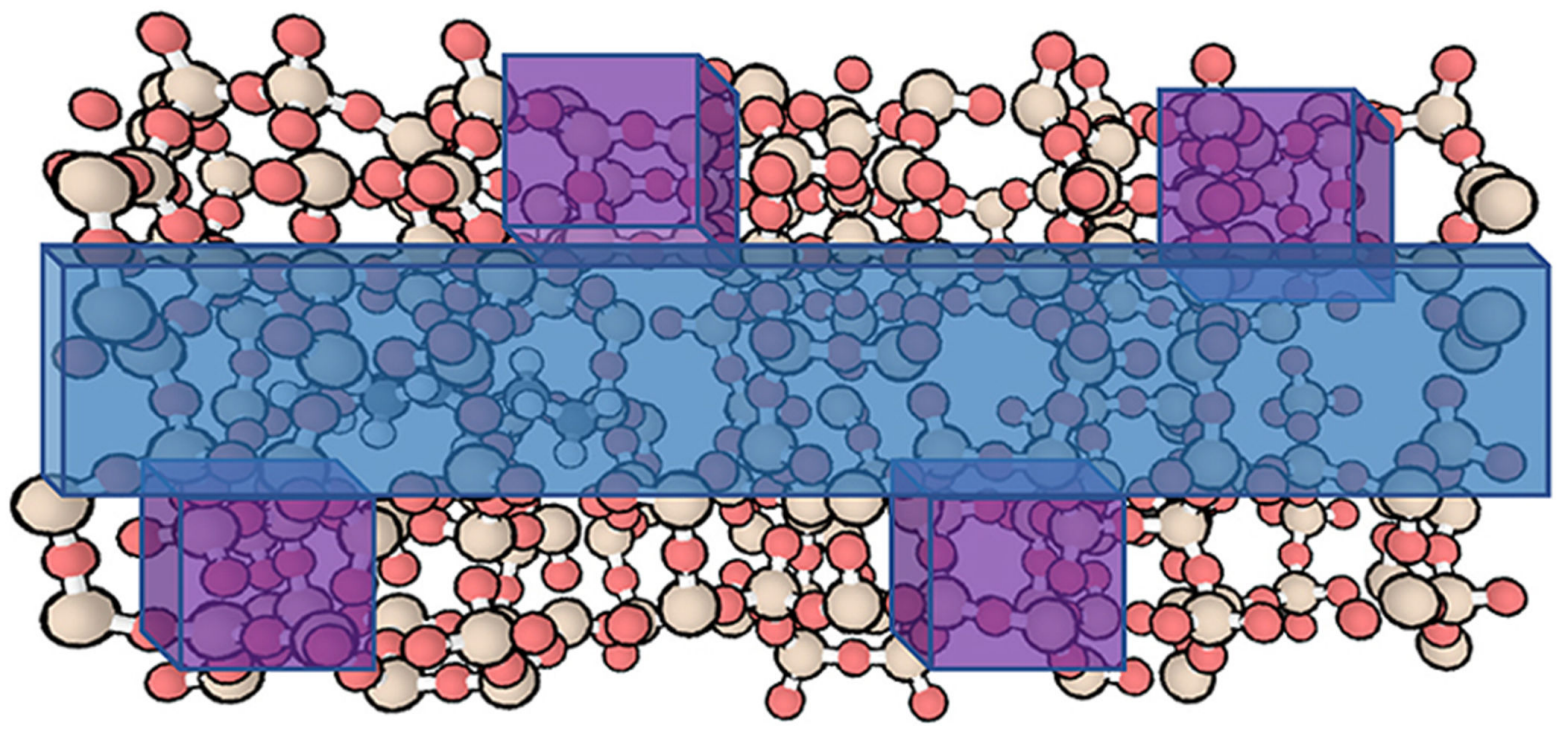
The H-ZSM-5 zeolite framework with the straight pore (blue) and sinusoidal zig-zag pores (purple) with the promoter molecule adsorbed in the left-hand side of the straight pore. The simulations involve user interaction with the end carbon of the alkyl chain, guiding the molecule to the right through the straight pore. (For interpretation of the references to colour in this figure, the reader is referred to the Web version of this article.)

These simulations employed a 1 x 2 x 1 supercell containing 600 atoms and periodic boundary conditions. This maximized the size of the simulation cell without increasing the computational expense to the point where iMD-VR with DL_POLY was impractical. The structure of H-ZSM-5 consists of intersecting straight and sinusoidal 10-membered ring pores as shown in Fig. 6; there are twelve distinct Al locations within one unit cell that can act as the Brønsted acid sites [78]. It has been shown that Al preferentially occupies sites at pore intersections, so we chose here to incorporate a Brønsted acid site at the intersection of two channel pores [72,79,80].

In the zeolite framework, Si, Al, and O atoms are assigned formal charges of +4.00, +3.00, and –2.00 respectively. At the Brønsted acid site, fractional charges of –1.46 and + 0.43 are assigned to the O and H atoms, respectively and the harmonic bond-angle three-body potentials to describe the O–Si–O and O–Al–O triads were those of Schroder et al. [81] The interatomic two-body S–O, Al–O and O–O Buckingham potentials used for the zeolite were based on the work of Sanders et al. [82] and Catlow et al. [83] with cutoffs of 6.5 Å. The intramolecular parameters for methyl *n*-hexanoate were taken from Jorgensen et al. [34] and the intermolecular potential parameters between the zeolite and ester from work on adsorption of organic molecules at clay surfaces [84–86]. Previous work of Dennis Smither et al. has demonstrated the high adsorption capability of the promoter molecule at the Brønsted acid site [70]. Consequently, the starting structure in all cases was the promoter molecule adsorbed at the Brønsted acid site in the H-ZSM-5 zeolite structure. The initial stage of all simulations was an equilibration stage of 50 ps at 500 K in the canonical (*NVT*) ensemble, with a Langevin thermostat friction constant of 5 ps^–1^. After the equilibration run, a production run of 500 ps was carried out at 500 K. The timestep used was 1 fs and the atomic coordinates and the energy of the system were saved every 0.1 ps (100 steps) [87].

In the iMD-VR simulations, user interaction consisted of interacting with the end carbon of the alkyl chain of the promoter molecule and guiding it away from the Brønsted acid site through the straight pore. A variety of user interaction times were tried in turn: 1, 2, 4, 8 and 16 ps, each preceding a 500 ps production run. After the user interaction times, the final atomic positions, velocities, and forces were used as the starting point for a production run. The energy differences reported below are the difference in energy at each step compared to the average over the entire production run.

An initial production run of 500 ps of MD was conducted without any user bias. The fluctuation of the energy difference, and the mean-squared displacement of the centre of mass of the promoter molecule, can be found in the supplementary information. These data do not fluctuate significantly due to the strong adsorption of the promoter molecule at the Brønsted acid site.

**Fig. 7:**
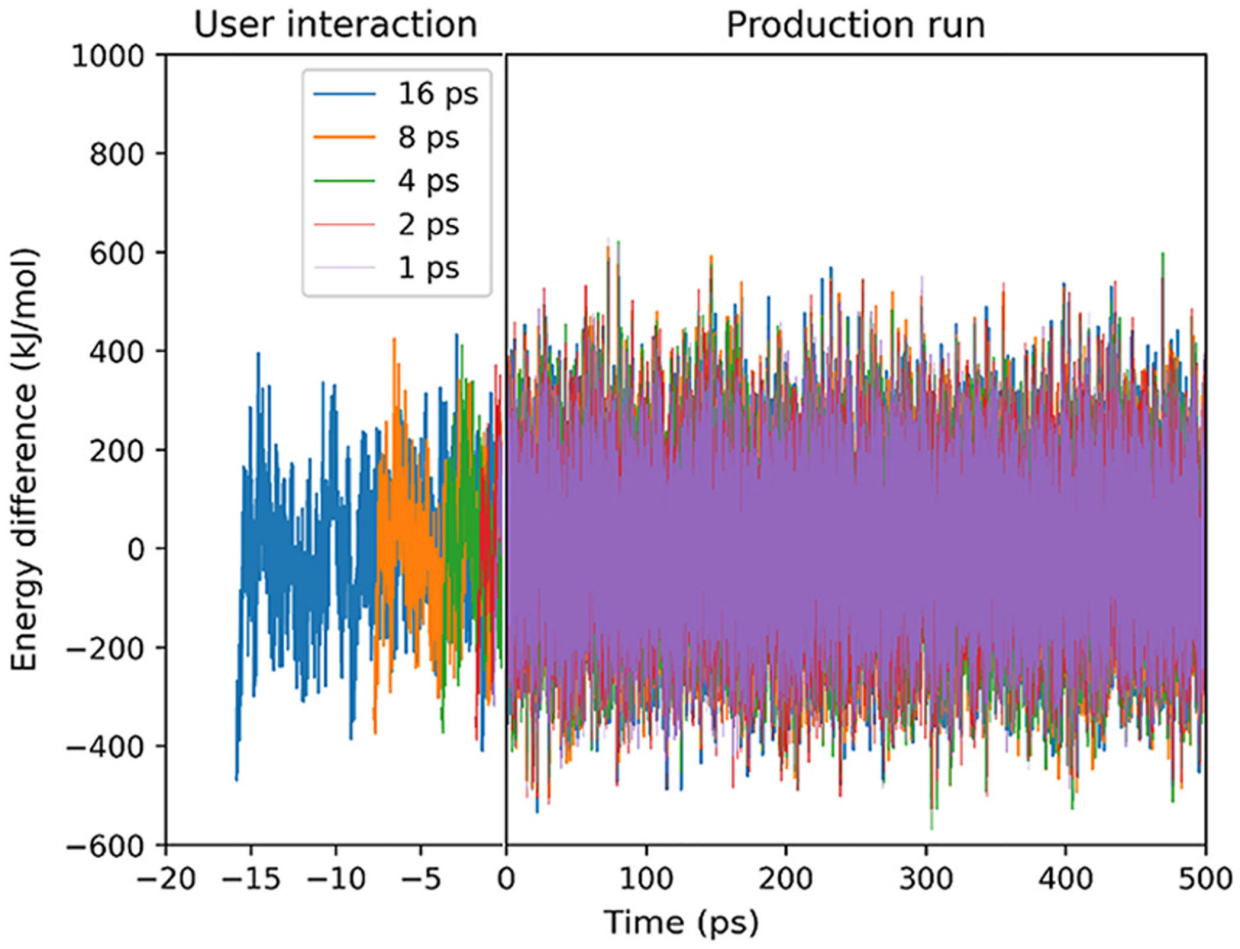
Fluctuations of the energy during the user-interaction times and the subsequent production runs. The left-hand side of the plot (negative times) shows the energy difference during the period in which the user interacted with methyl n-hexanoate, pulling it through the pore. The right-hand side of the plot shows the mean squared displacements in the subsequent 500 ps MD simulations.

No diffusion away from the site was observed over 500 ps, so iMD-VR was employed to guide the methyl *n*-hexanoate away from the Brønsted acid site and through the straight pore. This was achieved by interacting with the end carbon in the alkyl chain with handheld VR controllers and the application of a biasing force to guide the molecule through the straight pore. The deviation of the energy at each step from the average energy of each respective production run is plotted in Fig. 7. Fluctuations of energy between –600 and 600 kJ/mol are observed in the standard MD in Figs. SI–3 and during and after user interaction in iMD-VR in Fig. 7. As they are comparable with and without user interaction, iMD-VR is assumed to not perturb the system into an unrealistic state.

**Fig. 8:**
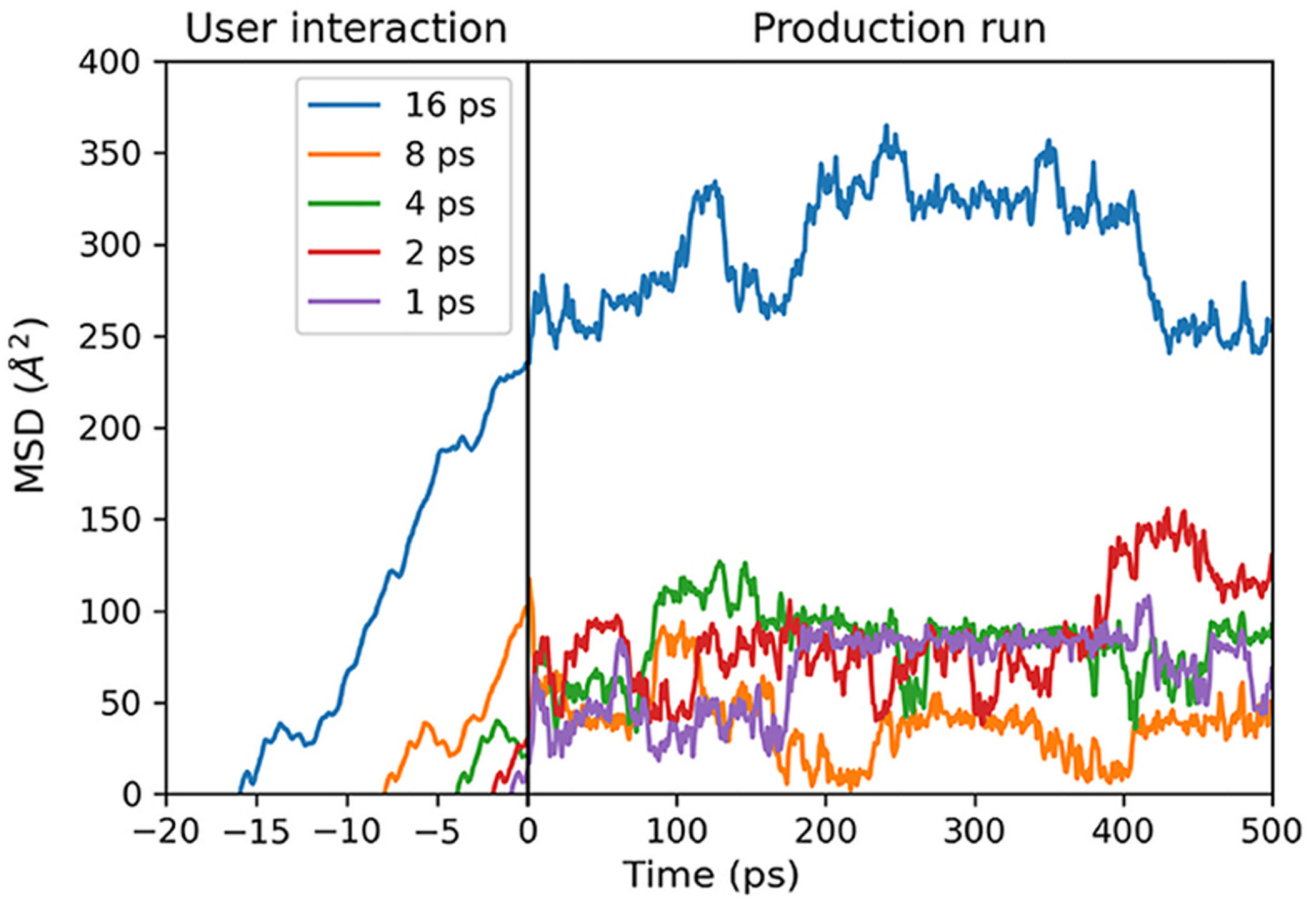
Mean-squared displacements of the centre of mass of methyl n-hexanoate in H-ZSM-5 zeolite along the straight pore. The left-hand side of the plot (negative times) shows the mean squared displacements during the period in which the user interacted with methyl n-hexanoate, pulling it through the pore. The right-hand side of the plot shows the mean squared displacements in the subsequent 500 ps MD simulations.

The mean-squared displacement of the promoter was monitored during and after user interaction in iMD-VR as shown in Fig. 8. The mean mean-squared displacements in the 500 ps production run after 1, 2, 4, 8, and 16 ps of user interaction were 66, 85, 83, 39, and 296 Å^2^, respectively. The ranges of the mean-squared displacement in the 500 ps production run after 1, 2, 4, 8, and 16 ps of user interaction were 90, 119, 105, 115, and 129 Å^2^, respectively. Although the mean value after 16 ps user interaction is much greater, the range is comparable to the other 500 ps production runs. This is due to similar diffusion behaviour in the production run in all cases, i.e., diffusion in and around the sinusoidal zig-zag pores. Nevertheless, the overall increased mean-squared displacement in the production run after 16 ps user interaction implies that the methyl *n*-hexanoate has overcome a significant bottleneck during the user interaction period.

**Fig. 9:**
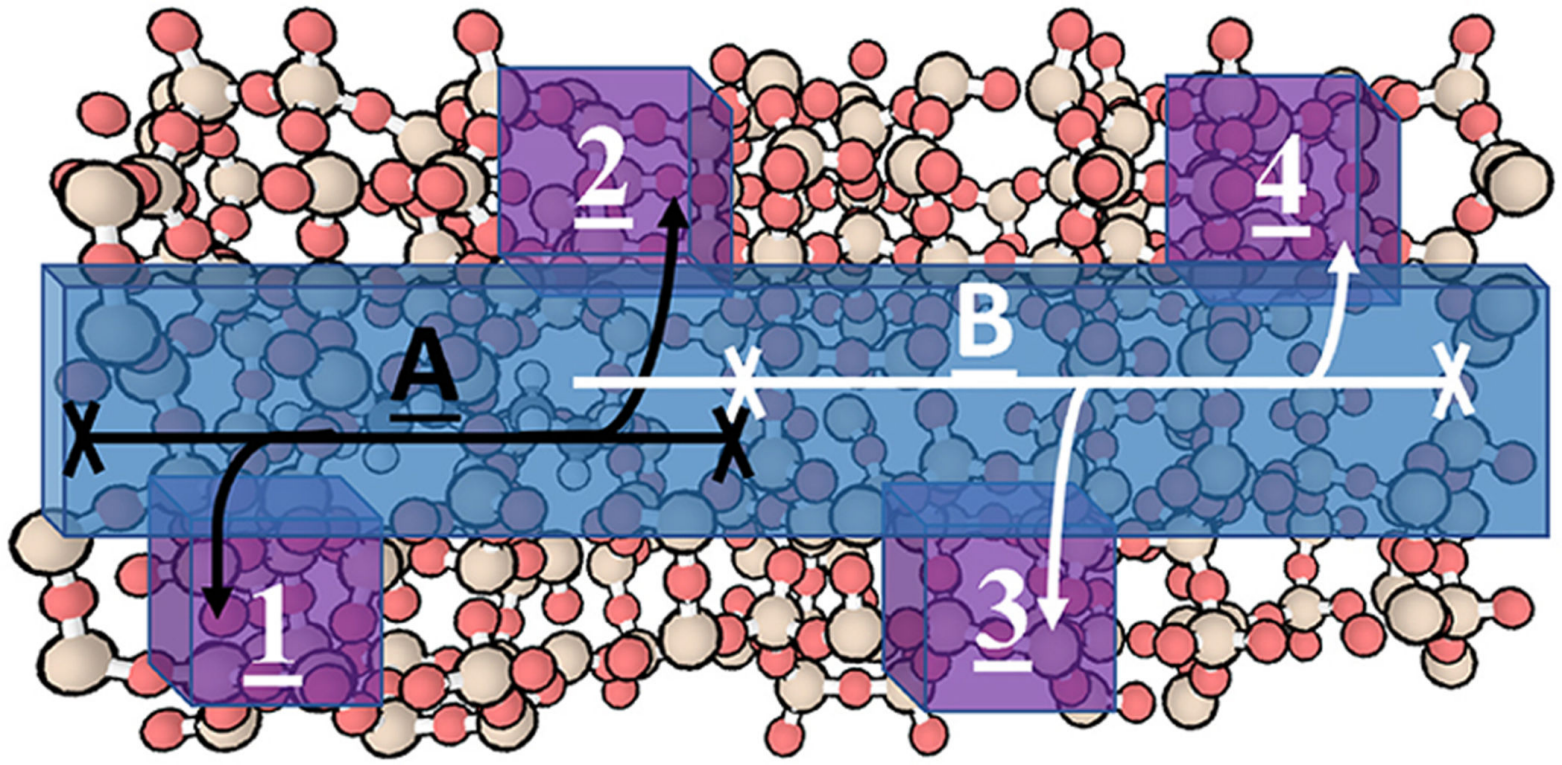
The methyl *n*-hexanoate molecule adsorbed in H-ZSM-5 zeolite with the pores of interest highlighted; the straight pore is highlighted in blue, and the sinusoidal zig-zag pores are highlighted purple and numbered 1 to 4. The two diffusion pathways are labelled A and B, and the steric barriers observed in iMD-VR are in the straight pore and occur; 1) between the zig-zag sinusoidal pores numbered 2 and 3, 2) After the zig-zag pore numbered 4 and 3) before the zig-zag pore numbered 1. (For interpretation of the references to colour in this figure, the reader is referred to the Web version of this article.)

The bottleneck exists in the straight pore between sinusoidal pores **2** and **3** indicated in Fig. 9. Of the simulations with 1, 2, 4, and 8 ps of user interaction the promoter molecule diffused along pathway **A**; only in 8 ps did the promoter reach the bottleneck. In the subsequent production run, the promoter molecule rapidly diffused back toward the Brønsted acid site. With 16 ps user interaction, the promoter passed the bottle-neck, moving from pathways **A** to **B**, hence the increased mean squared displacement.

The presence of a bottleneck in the straight pore hints at the existence of directed diffusion pathways more generally, i.e., preferential diffusion through the sinusoidal pores. The identification of such a bottleneck using iMD-VR shows how the technique may be used to identify important structural features related to catalysis in such complex systems. To make the approach more quantitative, an important future development will be the explicit calculation of the force exerted by the user.

## 4 Conclusions

We have demonstrated that iMD-VR is a potentially a valuable tool in materials chemistry both in teaching and in research; it enhances the visualisation experience over what is available from a rigid model or from the conventional trajectory playback in a molecular dynamics simulation.

We have used it here to probe the formation of defects and the mechanism of ion transport and fast ion conduction in lithium oxide, and to explore the transport of molecules within zeolites. iMD-VR allows the user an immersive experience in which they can apply their chemical intuition to manipulate structures and to sample relevant areas of an energy landscape which relate to a specific property. It provides a method to accelerate discovery processes, offers the potential to identify bottlenecks, and may allow rare events to be analysed on otherwise inaccessible timeframes. iMD-VR is thus a quick and efficient, if currently very approximate, accelerated dynamics technique as well as being a useful way of visualising, interpreting, and manipulating complex 3D structures, with potential applications in research (e.g. materials design and discovery) as well as teaching, including for groups of users working together in the same virtual environment.

## Supplementary Material

Multimedia component 1

## Figures and Tables

**Table 1:** Materials examined in iMD-VR, a brief description, and their respective URLs of the relevant videos. The videos show the user’s perspective in iMD-VR.

Video Index	URL	Description
**A1**	vimeo.com/408001520	Structure and dynamics of liquid argon
**A2**	vimeo.com/404944477	Effect of the choice of the thermostat on an SPC water model
**A3**	vimeo.com/408008421	Constraint dynamics used to calculate the potential of mean force for H_2_O – K^+^
**A4**	vimeo.com/408008637	Potassium capture by valinomycin
**A5**	vimeo.com/408009725	Pressure induced phase change in potassium chloride
**A6**	vimeo.com/429567164	The adsorption of formic acid on a calcite surface
**A7**	vimeo.com/408007873	The structure and dynamics of a perovskite solar cell
**A8**	vimeo.com/410994288	Fast ion conduction in silver iodide
**A9**	vimeo.com/410994555	Fast ion conduction in zirconia
**A10**	vimeo.com/408004650	Structure and dynamics of graphene
**A11**	vimeo.com/446795142	Structure and dynamics of a carbon nanoswitch
**A12**	vimeo.com/419861418	Transport of benzene through a ZSM-5 zeolite
**A13**	vimeo.com/493375346	Adsorption and migration of ion pairs on the (100) surface of barium oxide
**B14**	vimeo.com/410995001	Fast ion conduction in Li_2_O
**B15**	vimeo.com/456311091	Transport of methyl *n*-hexanoate through H-ZSM- 5 zeolite using iMD-VR

## Data Availability

All input files are available via the link in the abstract. The trajectory files for the lithium oxide and zeolite examples are also available via the same link.
