# Supplementary material for: Interactive molecular dynamics in virtual reality for modelling materials and catalysts
Source: J Mol Graph Model. Author manuscript; Available in PMC 2024 Nov 5. (PMC7616772; doi:10.1016/j.jmgm.2023.108606)
Supplement: Multimedia component 1 [file EMS199416-supplement-Multimedia_component_1.pdf]

Supplementary information

In this section we present examples of videos that provide an indication of the range of systems that can be visualised/studied by iMD_VR. The examples presented include DL_POLY tutorial exercises (denoted *), as well as other relevant systems. Table 1 lists all the videos available, each of which show the visualisation in iMD-VR from the user's point of view.

A1 - Studying the structure and dynamics of liquid argon *

In 1971 liquid argon was one of the first liquids to be studied by MD [94]. In this example fifteen thousand atoms of liquid argon are simulated at 850 K in the micro-canonical ensemble (NVE) in this example. The bulk liquid behaviour can be examined using iMD-VR (https://vimeo.com/408001520) which highlights the dynamical motion of the atoms.

A5 - Induced phase change in potassium chloride *

At ambient conditions, potassium chloride (KCl) has the face-centred cubic rock-salt structure but under compression at high pressure, the structure alters to a denser cubic structure, that of CsCl, with higher anion and cation coordination numbers. In this example, two hundred and sixteen atoms of KCl in the cubic phase are simulated at 200 K at 100 kbar inducing a phase change (https://vimeo.com/408009725). The video shows the mechanism of the transition via a rhombohedral distortion – the Buerger mechanism [95].

A6 - Study of the absorption of formic acid on a calcite surface *

Formic acid absorbed on a calcite surface is simulated at 10 K in the canonical ensemble. Formic acid (and acetic acid) are the most abundant monocarboxylic acids in the ambient atmosphere; the uptake and long-range transport of them by minerals such as calcite play an important role in global climate and regional environments [96]. In this example iMD-VR is used to guide a formic acid molecule towards specific adsorption sites on the calcite surface (https://vimeo.com/429567164).

A8 & A9– Fast ion conduction in silver iodide* & zirconia.

The high temperature α-phase of silver iodide is a fast ion conductor (https://vimeo.com/410994288). The larger iodide anions form a rigid body centred cubic lattice, and the smaller, highly mobile silver cations occupy pseudo-tetrahedral sites amongst the anions and behave as a quasi-liquid. When zirconia, ZrO_2, is doped with a lower valent cation, such as yttria or calcium, anion vacancies are produced (https://vimeo.com/410994555) to satisfy charge neutrality. The anion vacancies readily migrate readily through the crystal, leading to the high ionic conductivity. In both cases, the advantage of using iMD-VR, as discussed in the main text, is the potentially straightforward manner of investigating transport mechanisms.

A10 – Simulation of graphene using a molecular mechanics potential.

A graphene sheet with one hundred and sixty atoms is simulated at 50 K in the micro-canonical ensemble. In the main text iMD-VR with DFTB+ is used to study the formation of the Stone-Wales defect in graphene. Here a classical simulation of graphene is presented (https://vimeo.com/408004650); the video demonstrates the long-range rippled deformation that can be achieved and observed in iMD-VR. Furthermore, the magnitude of the deformation can be tuned through the interaction scaling factor on the hand-held controllers.

A11 - The structure and dynamics of a carbon nanoswitch *

Two carbon nanotubes, one within the other, make up a carbon nanoswitch and are simulated in this example at 295 K in the micro-canonical ensemble (NVE) (https://vimeo.com/446795142). Visualisation of the shuttling behaviour of the nanotubes leading to its switch-like properties is examined in iMD-VR.

A12 –The diffusion of rigid body benzene through a zeolite framework *

In this example, a zeolite, a complex cage structure of silica and alumina, and rigid body benzene are simulated at 500 K in the canonical ensemble (NVT) (https://vimeo.com/419861418). The ease in which a user can analyse different pathways for small molecules in zeolites using iMD-VR is demonstrated in this example.

A13 – Surface diffusion on a barium oxide surface

The (100) barium oxide surface is simulated at 1000 K in the canonical (NVT) ensemble (https://vimeo.com/493375346). A barium oxide pair is placed above the surface in VR and diffuses across the surface. The ion pair diffuses by an exchange mechanism, rather than hopping across the surface. This is consistent with the detailed hyperdynamics simulations of Harris et al. [97]; the advantage of the iMD-VR here, as for Li_2O discussed in the main text, is the immediacy and straightforward way of establishing the transport mechanism.

Supplementary information for the Li_2O example in the main text:

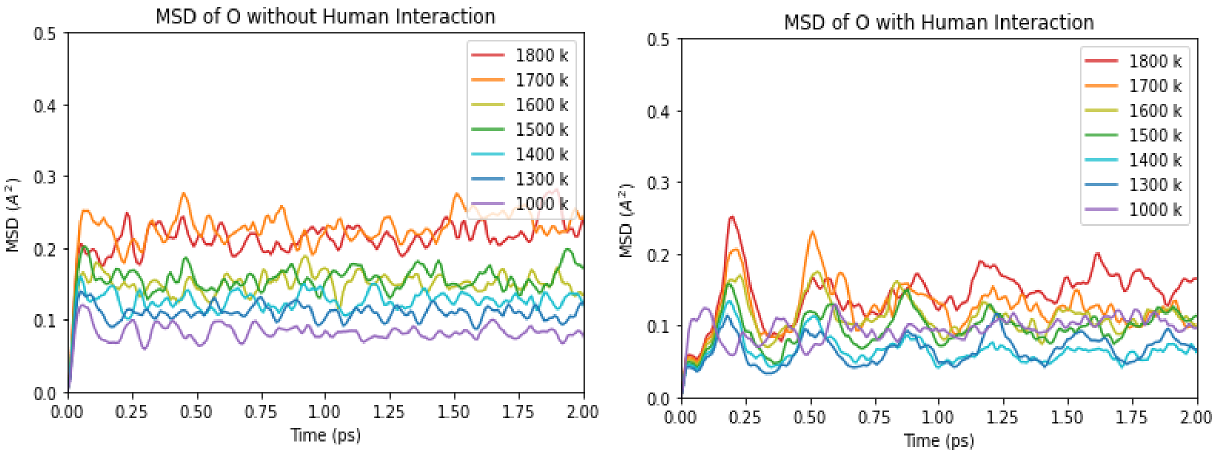

Figure SI-1-(Left) The mean squared displacement of the oxygen ions at temperatures between 1000 and 1800 K without any user bias interaction. (Right) The mean squared displacement of the oxygen ions at temperatures between 1000 and 1800 K over 2 ps of user bias interaction time.

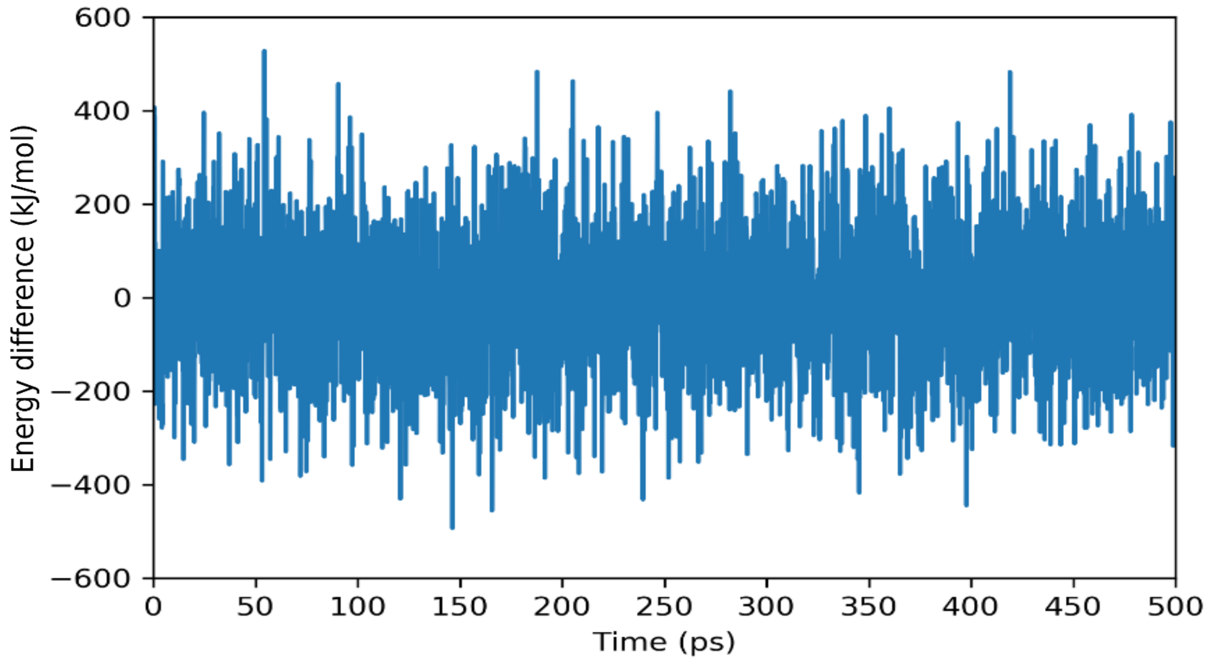

Figure SI-2 Fluctuation of the energy difference of the promoter molecule and H-ZSM-5 system during the production run of 500 ps of standard MD with no user interaction. The energy difference is the difference in the total system energy compared to the average total system energy calculated over the entire 500 ps production run.

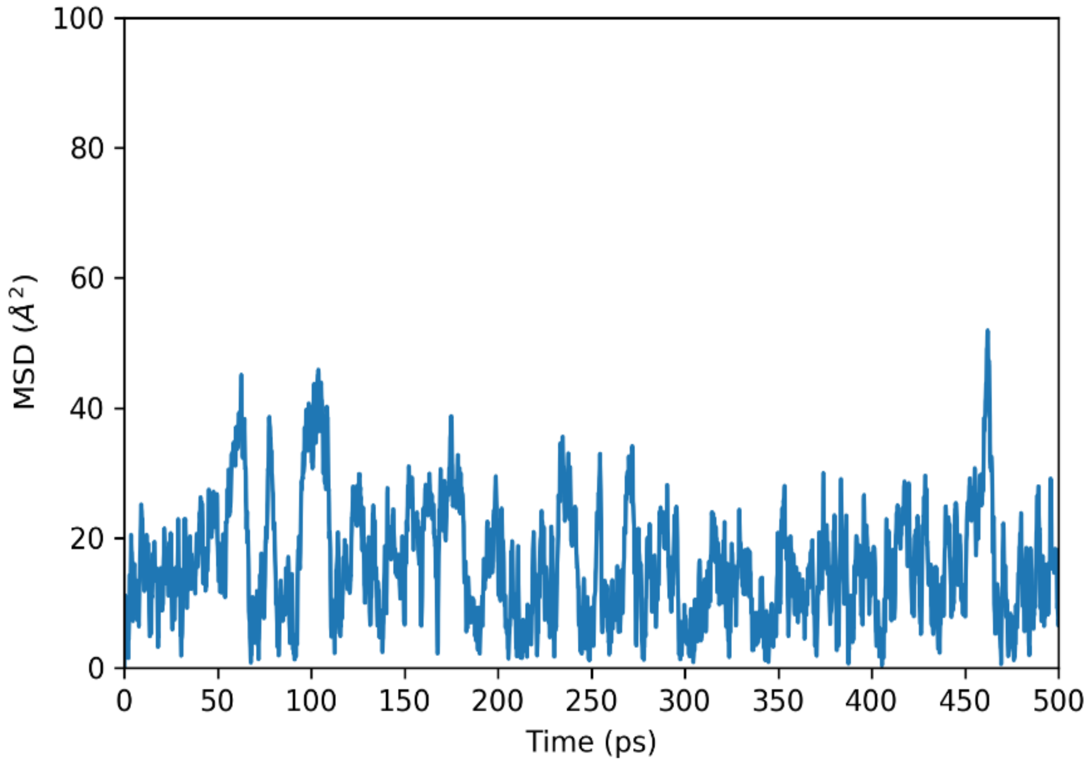

Figure SI-3 Mean-squared displacement of the centre of mass of the promoter molecule within H-ZSM-5 during the production run of 500 ps of standard MD without user interaction.
